# Landscape factors and allochthonous congeneric species influence *Callithrix aurita* occurrence in Brazilian Atlantic Forest remnants

**DOI:** 10.1002/ece3.9968

**Published:** 2023-04-07

**Authors:** Natasha Grosch Loureiro, Vanessa de Paula Guimarães‐Lopes, Flávio Henrique Guimarães Rodrigues, Rodrigo Lima Massara

**Affiliations:** ^1^ Laboratório de Ecologia de Mamíferos, Departamento de Genética, Ecologia e Evolução, Instituto de Ciências Biológicas Universidade Federal de Minas Gerais Av. Antônio Carlos 6627, Pampulha, CP 486 Belo Horizonte MG CEP 30270‐901 Brazil; ^2^ Programa de Pós‐graduação em Ecologia, Conservação e Manejo da Vida Silvestre, Laboratório de Ecologia de Mamíferos, Departamento de Genética, Ecologia e Evolução, Instituto de Ciências Biológicas Universidade Federal de Minas Gerais Av. Antônio Carlos 6627, Pampulha, CP 486 Belo Horizonte MG CEP 30270‐901 Brazil; ^3^ Programa de Pós‐graduação em Ecologia, Conservação e Manejo da Vida Silvestre, Laboratório de Ecologia e Conservação, Departamento de Genética, Ecologia e Evolução, Instituto de Ciências Biológicas Universidade Federal de Minas Gerais Av. Antônio Carlos 6627, Pampulha, CP 486 Belo Horizonte MG CEP 30270‐901 Brazil

**Keywords:** alien species, biodiversity hotspot, *Callithrix aurita*, land use and cover, species management, surrounding matrix

## Abstract

The buffy‐tufted‐ear marmoset (*Callithrix aurita*) is a small primate endemic to the Brazilian Atlantic Forest biome, and one of the 25 most endangered primates in the world, due to fragmentation, loss of habitat, and invasion by allochthonous *Callithrix* species. Using occurrence data for *C. aurita* from published data papers, we employed model selection using Akaike Information Criterion corrected for small samples and cumulative AICc weight (*w*_+_) to evaluate whether fragment size, distance to fragments with allochthonous species, altitude, connectivity, and surrounding matrices influence the occurrence of *C. aurita* within its distributional range. Distance to fragments with *C. jacchus* (*w*_+_ = 0.94) and nonvegetated areas (*w*_+_ = 0.59) correlated negatively with *C. aurita* occurrence. Conversely, the percentage of agriculture and pasture mosaic (*w*_+_ = 0.61) and the percentage of savanna formation (*w*_+_ = 0.59) in the surrounding matrix correlated positively with *C. aurita* occurrence. The findings indicate that *C. aurita* is isolated in forest fragments surrounded by potentially inhospitable matrices, along with proximity of a more generalist and invasive species, thereby increasing the possibility of introgressive hybridization. The findings also highlighted the importance of landscape elements and allochthonous congeneric species for *C. aurita* conservation, besides indicating urgency for allochthonous species management. Finally, the approach used here can be applied to improve conservation studies of other endangered species, such as *C. flaviceps*, which is also endemic to the Brazilian Atlantic Forest and faces the same challenges.

## Graphical abstract

*Callithrix aurita* is isolated in forest fragments surrounded by potentially inhospitable matrices, along with the proximity of a more generalist and invasive species, thereby increasing the possibility of introgressive hybridization. The approach used here can be applied to improve conservation studies of other endangered species, such as *C. flaviceps*, which is also endemic to the Brazilian Atlantic Forest and faces the same challenge.

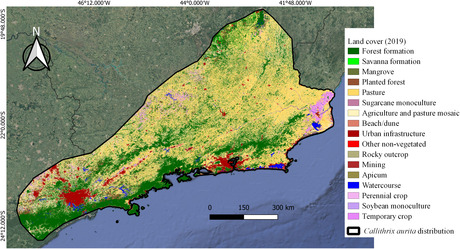

## 1 INTRODUCTION

The conversion of native habitats into anthropic landscapes, and the accompanying habitat fragmentation and loss, poses a challenge for primate conservation efforts (Estrada et al., 2017; Foley et al., 2005). These processes change environmental integrity (e.g., heterogeneity and structure previous to human activities), on a landscape scale and can increase isolation and reduce large extensions of habitat into smaller fragments, which are immersed in altered matrices (Andrén, 1994; Hanski, 2015). The effects of habitat loss on species are potentiated when they are associated with human activities, such as logging, agriculture, hunting, and trafficking (Estrada et al., 2017). Anthropogenic activities that promote fragmentation can also isolate protected areas and increase susceptibility to invasion by allochthonous species (Spear et al., 2013), which represent another major threat to species conservation (Butchart et al., 2010).

Neotropical primates constitute an excellent model to evaluate the effects of landscape changes since these animals are arboreal and, in general, ecologically less flexible than terrestrial mammals in terms of vegetation cover loss (Arroyo‐Rodríguez & Mandujano, 2009; Isaac & Cowlishaw, 2004). Studies show that primate ecology such as distribution, diet, home range, and even social organization can be affected at several levels (Arroyo‐Rodríguez et al., 2008; Cristóbal‐Azkarate & Arroyo‐Rodríguez, 2007; Zunino et al., 2007). Although some generalist primate species can perform landscape supplementation (Dunning et al., 1992), which means they can search and acquire resources in neighboring fragments or in the matrix itself (Asensio et al., 2009), landscape attributes directly influence the establishment of their populations in forest fragments (Arroyo‐Rodríguez et al., 2008).

Soon after changes occur in forest cover, such as selective logging, primates tend to be randomly distributed in the landscape forest fragments. However, throughout the process, they end up colonizing only fragments that meet their environmental requirements (Chapman et al., 2003; Marsh, 2003). Such requirements may include a minimum fragment size according to their home range, and food resources. Some of these requirements are well‐known for a few primate species (Arroyo‐Rodríguez & Mandujano, 2009), but are relatively unknown for other species. Determining these requirements, and the consequent suitability of habitats, is important to understand the occupation (Arroyo‐Rodríguez et al., 2008) and occurrence (Hoffman & O'Riain, 2012; Silva et al., 2015) of primate populations and, therefore, the development of plans for their conservation (e.g., Andrew & Ustin, 2010). Understanding such factors is especially important for species that inhabit areas with high levels of fragmentation (Robbins & McNeilage, 2003), as is the case of *Callithrix* species in the Atlantic Forest.

The conservation status of the genus *Callithrix* is worsened by hybridization between species, which generates fertile offspring with intermediate characteristics (Malukiewicz et al., 2015). This is one of the greatest current threats to the conservation of buffy‐tufted‐ear marmoset (*Callithrix aurita*) (Carvalho et al., 2018), a marmoset endemic to the Brazilian Atlantic Forest in the states of Minas Gerais, Rio de Janeiro, and São Paulo. As a biome characterized by high species diversity and a high degree of endemism, the Atlantic Forest is one of the main biodiversity hotspots in the world, despite being highly fragmented with only 12.4% of the original extension remaining (SOS Mata Atlântica, 2019). Habitat loss and fragmentation, allied with the co‐occurrence of congeneric invasive species, are the main extinction threats for *C. aurita* (Malukiewicz et al., 2021). The International Union for Conservation of Nature (IUCN) Red List categorizes *C. aurita* as Endangered (Melo et al., 2021). Furthermore, the species is among the 25 most endangered primates of the world (Schwitzer et al., 2019) and is included in the National Action Plan for the Conservation of Atlantic Forest Primates and the Collared Sloth (PAN PPMA) (ICMBio/MMA, 2018). This species has more specialized characteristics regarding feeding behavior in comparison with *C. penicillata* and *C. jacchus*, such as having less capacity of tree‐gouging to eat gum, which also restricts its distribution (Rylands et al., 2009). Its home range encompasses up to 35.3 ha (Corrêa et al., 2000), despite occurring in forest fragments with as low as 3 ha (Oliveira, 2012), and is generally found at altitudes above 500 m (Rylands & Faria, 1993).

The two most generalist species of the genus are *C. jacchus* and *C. penicillata*, the former originally ocurred in the Caatinga and in part of the Atlantic Forest in Northeast Brazil and the latter in the Cerrado (Hershkovitz, 1977; Raboy et al., 2008). However, the distributions of these species have expanded due to introduction processes in other regions of Brazil (Oliveira & Grelle, 2012; Rylands, 1993). They are extremely flexible ecologically, and so are categorized as Least Concern by the IUCN (Bezerra et al., 2018; Bicca‐Marques et al., 2018). The use of gummivory (i.e., a diet based on plant exudates) with greater intensity makes them more generalist and facilitates their adaptation to new areas (Abreu et al., 2016; Vilela & Del‐Claro, 2011). This factor also influences their home ranges, which reach only 0.5 ha for *C. jacchus* (Stevenson & Rylands, 1988) and 2.5 ha for *C. penicillata* (Fonseca & Lacher, 1984).

Few studies have shown landscape factors as predictors of the occurrence of *Callithrix* species (e.g., Flesher, 2015), especially on a regional scale. For *C. aurita* specifically, the literature is scarce (e.g., Silva et al., 2015). In this context, this work aimed to evaluate whether landscape elements, such as patch attributes and the occurrence of allochthonous congeneric species, influence the occurrence of *C. aurita* within its distribution. We hypothesized that the probability of occurrence of *C. aurita* would be: (1) positively influenced by forest fragment altitude, area, connectivity, and distance to allochthonous species (Arroyo‐Rodríguez & Mandujano, 2009; Carvalho et al., 2018; Corrêa et al., 2000; Malukiewicz, 2019; Palacios, 2018; Rylands & Faria, 1993); (2) positively influenced by a surrounding matrix of forest (i.e., forest formation; Arroyo‐Rodríguez & Mandujano, 2009); and (3) negatively influenced by a surrounding matrix of other categories of matrices (i.e., savanna formation, pasture, agriculture and pasture mosaic, nonvegetated, flooded areas, planted forest and agriculture; Arroyo‐Rodríguez & Mandujano, 2009).

## 2 MATERIALS AND METHODS

### 2.1 Study area

**FIGURE 1:**
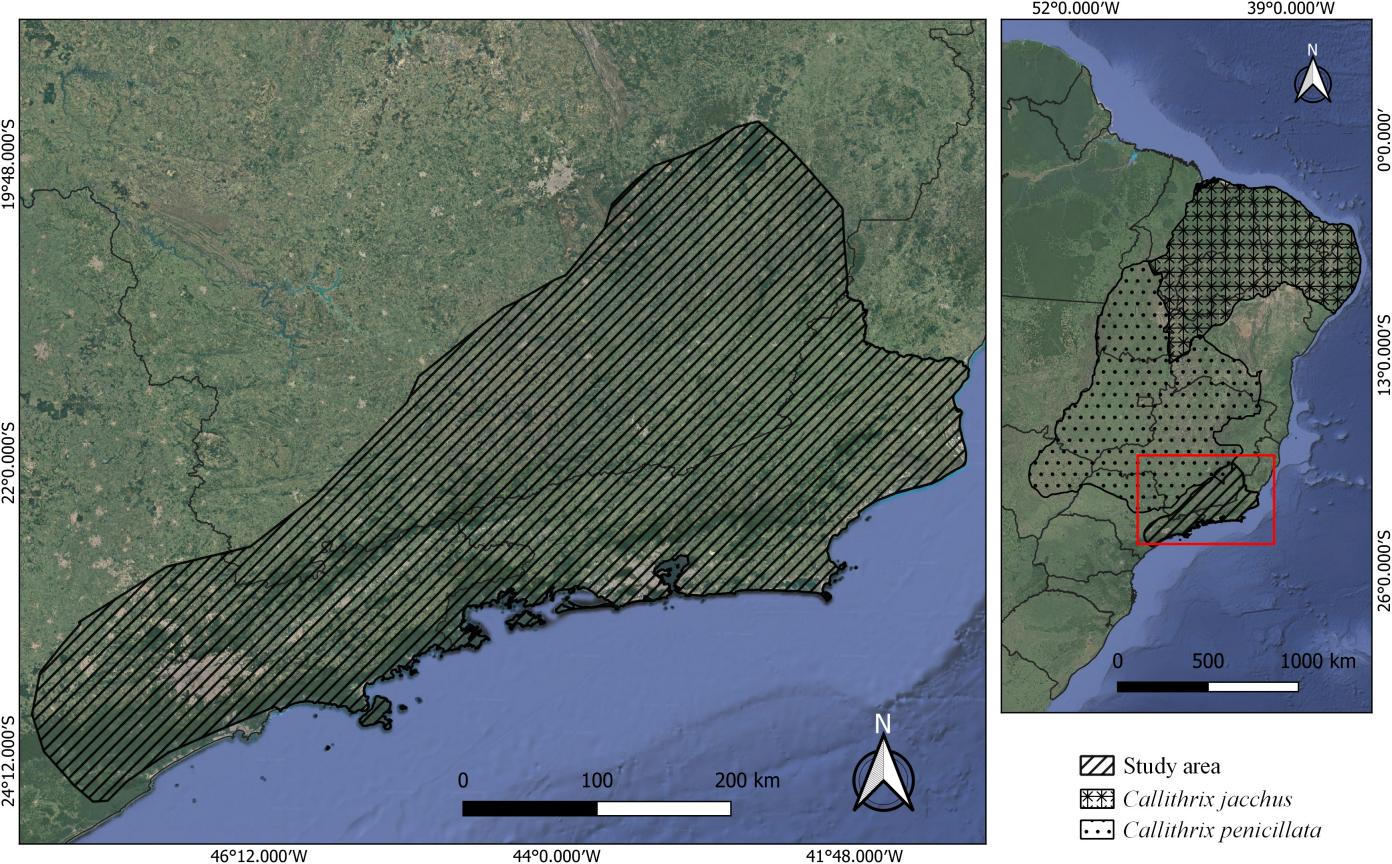
Map of the study area (hatched) delimited by the distribution of *Callithrix aurita* in Southeast Brazil (adapted from Melo et al., 2021). The original distribution areas of the allochthonous congeneric species are represented by the other filled areas.

The study was carried out within the original distribution of *C. aurita*, adapted based on the map provided by the IUCN (Melo et al., 2021), including Atlantic Forest regions of Brazil in the states of Minas Gerais, Rio de Janeiro, and São Paulo (Figure 1). This area harbors about 315 protected areas, including strategic locations for the preservation of Atlantic Forest (DATAGEO, 2018; IDE/SISEMA, 2019; INEA, 2016). The original distributions of *C. penicillata* and *C. jacchus* are located in other regions of Brazil and were possibly introduced into the study area and thus are considered allochthonous species (Figure 1). The natural dispersion of these species to the study area is considered unlikely, considering the existence of barriers such as large discontinuous areas (Cerqueira et al., 1998; Morais et al., 2008).

### 2.2 Occurrence data compilation

We used occurrence data from two data papers: “Atlantic‐primates” (Culot et al., 2019) and “Neotropical alien mammals” (Rosa et al., 2020). Only occurrence records of the species of interest (*C. aurita*, *C. jacchus*, and *C. penicillata*) in the study area, and collected between 1985 and 2019, were included. This time window was used due to overlap with the land use and cover image database available in Atlantic Forest Collection 5 on the MapBiomas project platform (MapBiomas, 2020). These raster files are based on Landsat satellite images, with 30‐m resolution. We excluded records collected in urban areas, in addition to those obtained by methods without empirical evidence (e.g., interviews). The remaining records were inserted into the QGIS software version 3.10.13 (QGIS, 2020) for checking the points and validation regarding overlap with the study area. In case of doubts about the geographic coordinates specified in the original worksheet, they were checked and, if necessary, corrected based on the coordinates explained in the referenced articles.

After filtering the data, 23 forest fragments containing records of occurrence of *C. aurita* were obtained and the same number of control fragments (i.e., without confirmed records of *C. aurita*) were randomly selected, for a total of 46 study fragments. Importantly, all control fragments were within the study area and belonged to Atlantic Forest areas.

### 2.3 Landscape metrics

Landscape analysis within the study area employed QGIS software (QGIS, 2020), GRASS GIS version 7.8.4 (GRASS, 2020), and Fragstats version 4.2 (McGarigal et al., 2015).

Taking into account that (1) the spatial configuration of the Atlantic Forest biome did not change much in the last 30 years (the peak of changes in the biome was between 1950 and 1960; Fonseca, 1985); (2) we had, in some cases, registers of the species of interest for the same area in different years; and (3) the life expectancy of *Callithrix* species can be up to 21 years (Nishijima et al., 2012), we assumed that patches that were occupied when the data were collected for the first time would remain occupied in the subsequent years. Additionally, because we had a limited number of registers of the species of interest, we could not make separate analyses including specific time intervals. Thus, for the subsequent analysis, we combined all the registers of *Callithrix* species and averaged along the years the landscape metrics, named fragment area, Euclidean Nearest Neighbor Distance (ENN), the Proximity Index (PROX), and the surrounding matrix variables (see details below).

The average area of each study fragment was calculated on Fragstats software (Table 1), with input of raster images of land use and cover of the Atlantic Forest of the MapBiomas project (MapBiomas, 2020). We averaged area values from the sampling year to 2019. We used the eight‐cell neighborhood rule, in which all eight adjacent pixels of the same class type are considered as members of the same feature, since the four‐cell rule is conservative and may underestimate values (Turner & Gardner, 2015).

**TABLE 1:** Mean (minimum and maximum) values for variables extracted from fragments with and without (control) confirmed *Callithrix aurita* occurrence, which were used as predictor variables to evaluate their effects on *C. aurita* occurrence in Southeast Brazil.

Variable	Control fragments (mean, minimum and maximum)	Fragments with occurrence of *C. aurita* (mean, minimum and maximum)
Average altitude	701.51 (9.02–1614.86 m)	879.96 (298.89–1375.55 m)
Euclidean distance to nearest neighbor	67.39 (56.79–164.91 m)	68.55 (56–115.12 m)
Average area size	3345.95 (4.41–43,166.01 ha)	38,337.78 (7.74–361,270.79 ha)
Proximity index	1761.9 (3–12,779.86)	8911.1 (6.41–68,490.53)
Minimum distance to *C. penicillata*	41.94 (7.97–133.9 km)	24.49 (0–60.5 km)
Medium distance to *C. penicillata*	243.12 (190.66–300.83 km)	262.63 (190.62–350.17 km)
Minimum distance to *C. jacchus*	67.81 (1.21–148.81 km)	32.13 (0.22–86.03 km)
Medium distance *C. jacchus*	255.87 (173.81–373.98 km)	260.22 (183.59–403.69 km)
Forest formation matrix	22.56 (0.89%–54.18%)	31.82 (12.13%–77.12%)
Savanna formation matrix	0.14 (0%–2.4%)	0.58 (0%–8.2%)
Planted forest matrix	1.33 (0%–6.85%)	2.54 (0.1%–11%)
Agriculture and pasture mosaic matrix	15.53 (6.35%–26.94%)	23.35 (5.15%–45.93%)
Pasture matrix	44.44 (9.46%–79.05%)	31.68 (7.88%–69.21%)
Nonvegetated areas matrix	4.18 (0.01%–30.1%)	2.26 (0.05%–10%)
Agriculture matrix	1.63 (0%–32.15%)	0.27 (0%–1.88%)
Flooded areas matrix	2.37 (0%–28.12%)	2.03 (0%–14.46%)

Minimum and average distances to fragment/fragments with the occurrence of allochthonous species (*C. penicillata* and/or *C. jacchus*) were calculated using the centroids (i.e., geometric center) of fragments (Table 1). The variable altitude, on the contrary, was the average altitude of each fragment. Altimetric data were extracted from a TOPODATA digital elevation model (INPE, 2008) with 30‐m resolution (Table 1). Both metrics were calculated on QGIS.

Two metrics, calculated on Fragstats, were used as a proxy to obtain data regarding connectivity: Euclidean Nearest Neighbor Distance (ENN) and the Proximity Index (PROX). Euclidean Nearest Neighbor Distance quantifies the Euclidean distance between the focal fragment and the fragments of the same class, based on the distance between the centroids of the two closest cells between fragments (McGarigal et al., 2015). The higher the ENN, the greater the isolation of a fragment compared with the others. The PROX metric (Gustafson & Parker, 1992) calculates, within a predefined search radius, the areas of fragments of the same class as the focal fragment, divided by proximity (i.e., edge‐to‐edge Euclidean distance from the focal fragment to the others). In this case, the search radius was defined as 1000 m, which corresponds to the average daily distance traveled by *C. aurita* (Corrêa et al., 2000). The higher the PROX value, the greater the presence of closer and more continuous fragments of the same class (McGarigal et al., 2015). An average was computed per sample fragment for both metrics, based on values from the sampling year to 2019 (Table 1).

**FIGURE 2:**
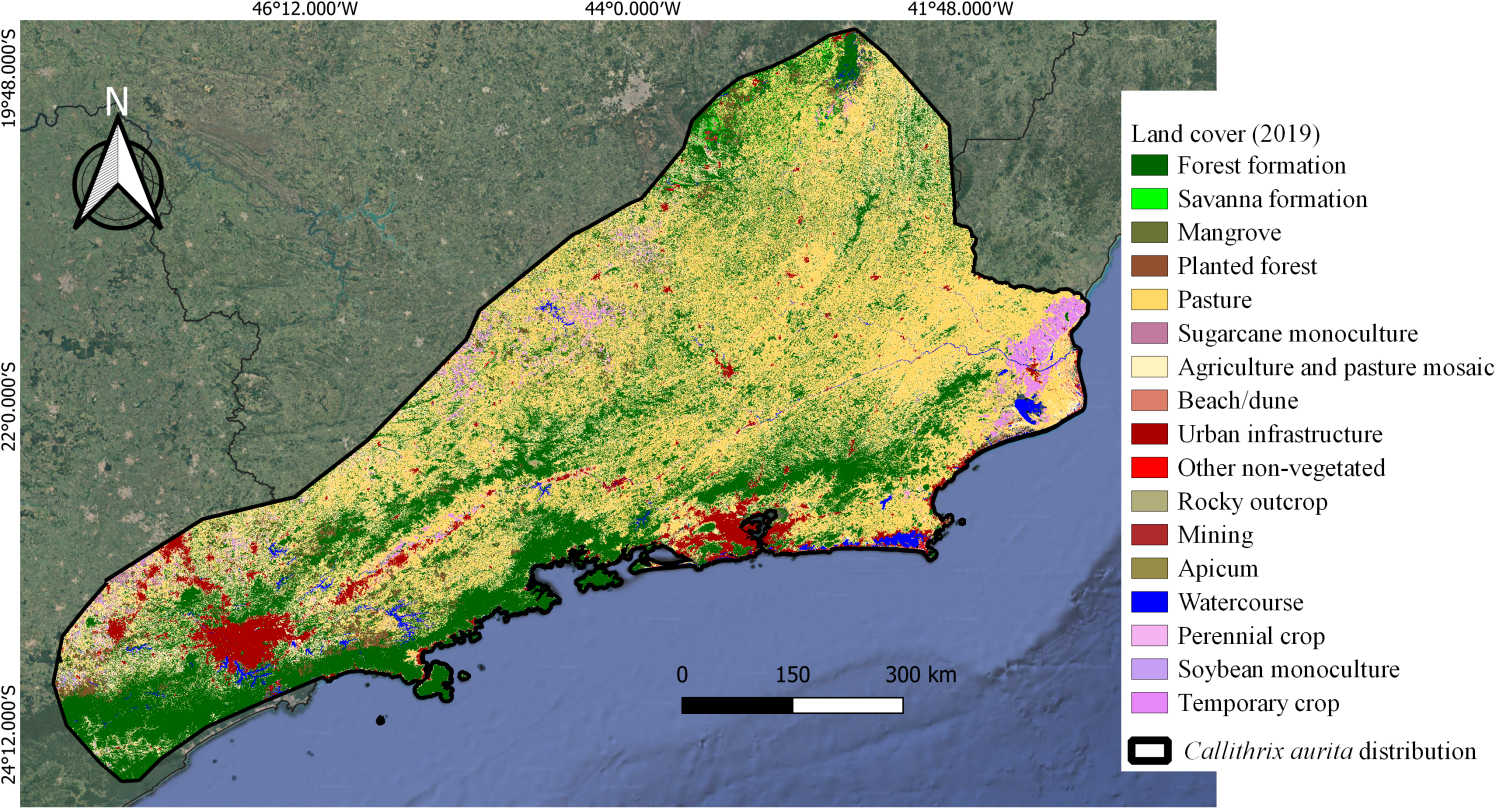
Map of land cover types found in the study area (distribution of *Callithrix aurita* adapted from Melo et al., 2021) relating to the year 2019, acquired on the Mapbiomas platform (MAPBIOMAS, 2020).

Finally, for the surrounding matrix, a 5 km buffer was created around each forest fragment. The buffers were submitted to GRASS GIS to obtain the area of each land use and cover class. For each fragment, this procedure was repeated covering every year between the sampling year and 2019. In the end, the average of each class was computed for each fragment and its percentage obtained. Seventeen land use and cover classes were detected: forest formation, savanna formation, mangrove, planted forest (i.e., tree species cultivated for commercial purposes, such as *Eucalyptus* sp. and *Pinus* sp.), pasture, sugarcane monoculture, agriculture and pasture mosaic, beach/dune, urban infrastructure, other nonvegetated (nonpermeable surfaces and exposed soil), rocky outcrop, mining, apicum (saline flooded area), watercourse, perennial crop, soybean monoculture, and temporary crop (Figure 2). For analysis purposes, and according to the ecology of *C. aurita*, these classes were categorized in order to unite similar types, resulting in eight matrix categories: forest formation, savanna formation, planted forest, agriculture and pasture mosaic, pasture, nonvegetated (urban infrastructure, other nonvegetated areas, rocky outcrops, beach/dune, and mining), agriculture (perennial crops, temporary crops, soybean monoculture, and sugarcane monoculture), and flooded areas (watercourses, mangroves, and apicum) (Table 1).

### 2.4 Data analysis

To assess whether landscape elements (i.e., patch attributes and landscape composition) and the presence of allochthonous species influence the probability of occurrence of *C. aurita* in forest fragments, we used generalized linear models (GLMs) with binomial distribution in the R environment version 4.0.5, using stats base package (R Core Team, 2021). The predictor variables used were average area of fragment, distance to fragments with occurrence of allochthonous species (for *C. penicillata* and for *C. jacchus*), average altitude of fragment, connectivity of fragment (ENN and PROX), and percentage of each matrix category (forest formation, savanna formation, planted forest, agriculture and pasture mosaic, pasture, nonvegetated, agriculture, and flooded areas) in the matrix surrounding the fragment (Appendix S1).

To test for strong correlations (*r* > .70) between predictor variables, we performed the Pearson's Correlation Coefficient test (Mukaka, 2012). As the test indicated a strong correlation between average distance to fragments with *C. penicillata* and average distance to fragments with *C. jacchus* (*r* = .80), only the minimum distance to fragment with *C. penicillata* and the minimum distance to fragment with *C. jacchus* were kept for subsequent analysis since they showed a weak correlation (*r* = .40). The variables average area of fragment and PROX also showed a strong correlation (*r* = .95), so the former variable was kept since there was another variable representing connectivity (ENN). To assess for overdispersion of the data (i.e., $\hat{c}$ > 1), we performed a bootstrap with 10,000 simulations of the most parameterized model using the DHARMa package version 0.4.4 (Hartig & Lohse, 2021) of the R environment. To investigate whether there was spatial autocorrelation in the model residuals, we performed a Moran's *I*‐test also using DHARMa package.

We used the Akaike Information Criterion corrected for small samples (AICc) (Burnham & Anderson, 2002), to select the most parsimonious models. We built a total of 1093 models representing all possible additive combinations of the predictor variables, limiting the models to up to four variables (Doherty et al., 2012). This strategy resulted in a balanced set of models (i.e., all the variables were represented equally in the same number of models) that allowed the interpretation of the cumulative AICc weight (*w*_+_) of each predictor variable (Burnham & Anderson, 2002). We considered variables to be determinant of the probability of occurrence of *C. aurita* to be those with *w*_+_ ≥ 0.50 (Berger & Barbieri, 2004). We performed these analyses in R version 4.0.5, using the MuMIn package version 1.43.17 (Barton, 2020; Appendix S2).

## 3 RESULTS

**FIGURE 3:**
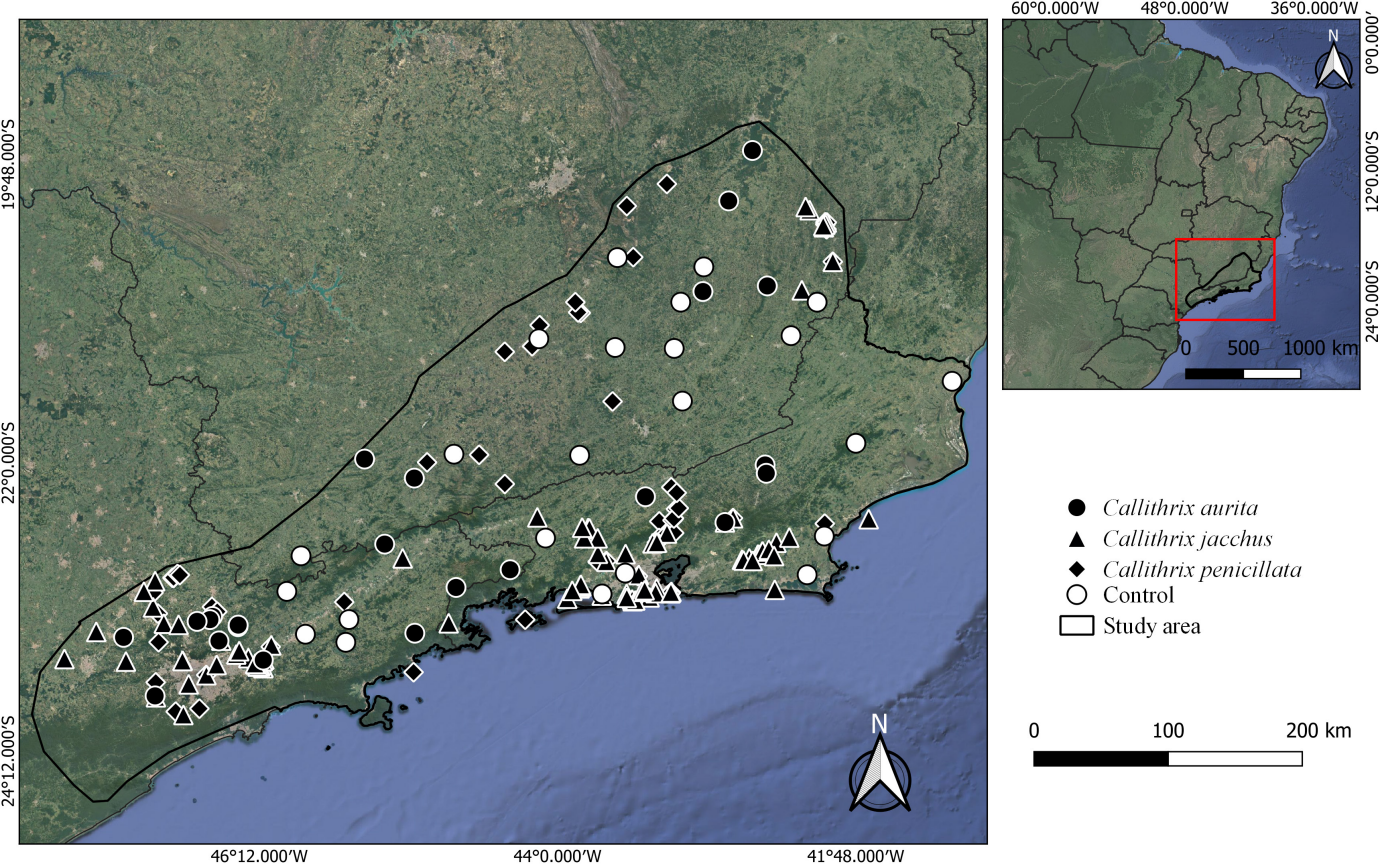
Map of records for *Callithrix aurita* (black ball), *C. penicillata* (lozenge), *C. jacchus* (triangle), and control fragments (without the confirmed presence of *C. aurita* white ball), located within the study area.

We compiled a total of 326 records of the genus *Callithrix*, with only 29 records of *C. aurita*, 95 of *C. penicillata*, and 202 of *C. jacchus* (Figure 3). Therefore, 297 records represent allochthonous congeneric species within the distribution area of *C. aurita*. The overdispersion test did not reveal any extrabinomial variation (i.e., overdispersion) in the data ($\hat{c}$ = 0.88, *p*‐value = .65). The spatial autocorrelation test showed independence of the spatial data (*I* = −0.03, *p*‐value = .82). The best‐ranked model (ΔAICc < 2) had an evidence weight of 22% (AICc weight = 0.22) and contained variables of allochthonous species presence and the surrounding matrix (percentage of agriculture and pasture mosaic, savanna formation, and nonvegetated). The variable average area of fragment was not present in the most parsimonious model and not even among the 10 best models, whereas the variable ENN appeared only in the seventh best model (Table 2).

**TABLE 2:** Model selection results for the 10 most parsimonious models of probability of *Callithrix aurita* occurrence as a function of minimum distance to *C. jacchus*; average altitude (ALT); minimum distance to *C. penicillata*; surrounding matrix with agriculture and pasture mosaic (MOS), savanna formation (SAV), non‐vegetated (NVEG), forest formation (FOF) and pasture (PAS); and Euclidean distance from nearest neighbor (ENN).

Models	AICc	ΔAICc	wAICc	df	Log‐likelihood	*R* ^2^
*C. jacchus* + MOS + SAV + NVEG	45.13	0	0.22	5	−16.815	.64
*C. jacchus* + MOS + FOF + SAV	47.55	2.42	0.07	5	−18.027	.60
*C. jacchus* + MOS + SAV + ALT	48.87	3.74	0.03	5	−18.686	.58
*C. jacchus* + SAV + NVEG + ALT	49.23	4.1	0.03	5	−18.864	.57
*C. jacchus* + MOS + NVEG	49.35	4.22	0.03	4	−20.189	.53
*C. jacchus* + SAV + ALT	49.41	4.28	0.03	4	−20.218	.53
*C. jacchus* + ENN + SAV + ALT	49.97	4.84	0.02	5	−19.236	.56

*Note*: AICc represents the ranking value for each model; ΔAICc represents the difference between the AICc value of a given model compared to the highest‐ranked model, wAICc (weight of AICc) represents the evidence weight of each model; and df the number of degrees of freedom or number of *β* parameters estimated for each model. Log‐likelihood shows the goodness of fit for each model and *R* ^2^ is the coefficient of determination.

**FIGURE 4:**
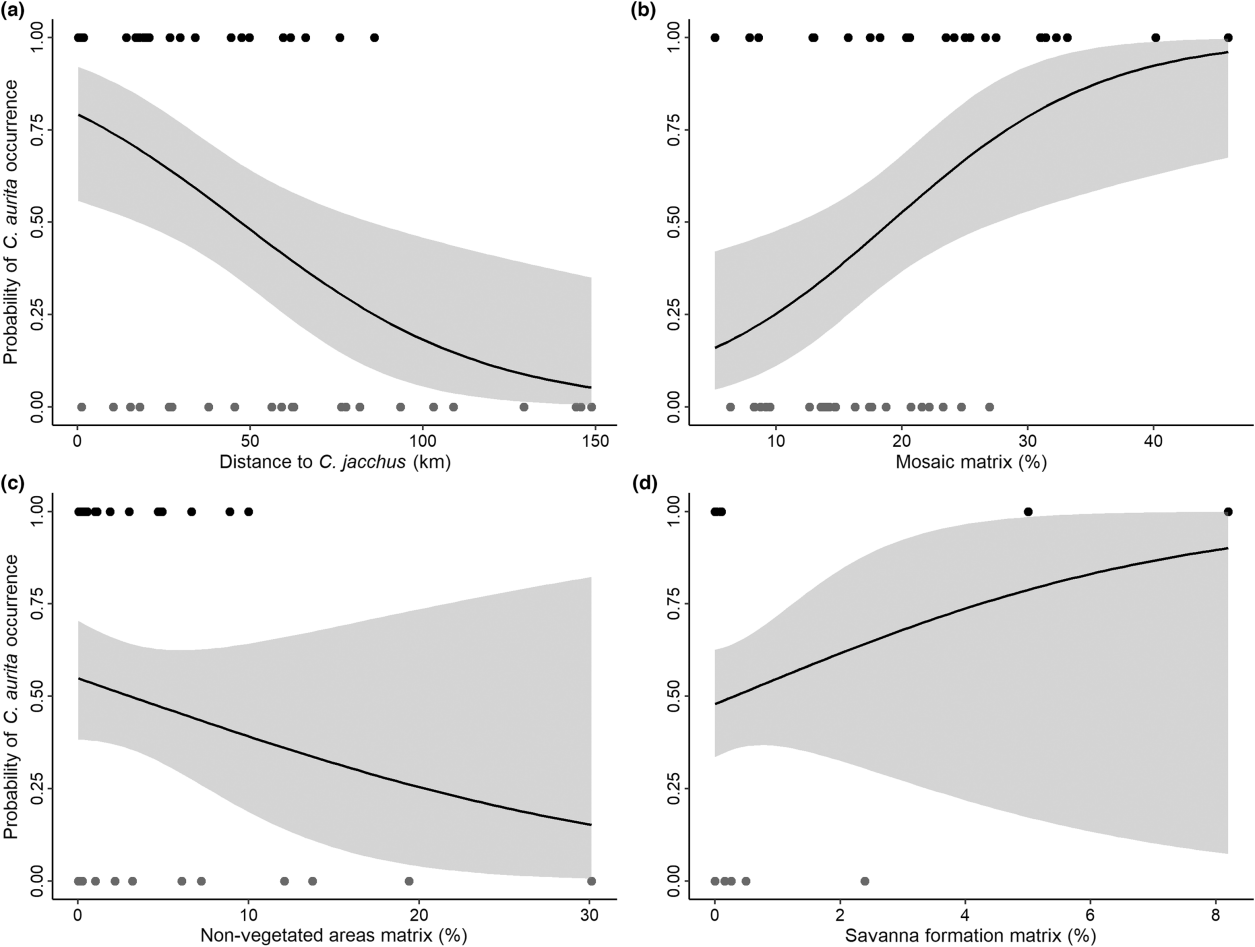
Effects of minimum distance to fragments with *Callithrix jacchus* (a), and percentages of agriculture and pasture mosaic (b), nonvegetated (c), and savanna formation (d) in the surrounding matrix, on the probability of *C. aurita* occurrence in forest fragments that occur within the species' distribution in Southeast Brazil. Black dots represent fragments with confirmed occurrence of *C. aurita* while gray dots represent those without (control). The gray range represents the confidence intervals.

The variable with the greatest influence on the probability of occurrence of *C. aurita* was minimum distance to fragment with *C. jacchus* (*w*_+_ = 0.94; Table 3), followed by three variables referring to open and/or anthropic matrix categories in the surrounding matrix: agriculture and pasture mosaic percentage (*w*_+_ = 0.61; Table 3), nonvegetated percentage (*w*_+_ = 0.59; Table 3) and savanna formation percentage (*w*_+_ = 0.59; Table 3). Minimum distance to fragment with *C. jacchus* (Figure 4) and nonvegetated percentage (Figure 4) were negatively correlated with *C. aurita* occurrence, while agriculture and pasture mosaic percentage and savanna formation percentage were positively correlated with *C. aurita* occurrence (Figure 4).

**TABLE 3:** Cumulative weight value of AICc (*w*_+_) for each predictor variable evaluated as a possible influencer of the probability of *Callithrix aurita* occurrence.

Variable	*w*_+_	*β* parameters		
		Estimate	Lower CI 95%	Higher CI 95%
Minimum distance to *C. jacchus*	0.94	−0.05	−0.09	−0.02
Agriculture and pasture mosaic matrix	0.61	17.4	5.44	33.50
Nonvegetated areas matrix	0.59	−25.8	−55.23	−6.82
Savanna formation matrix	0.59	90.05	16.13	314.61
Average altitude	0.25	3 × 10^−3^	3 × 10^−4^	6 × 10^−3^
Forest formation matrix	0.21	5.91	1.20	11.86
Pasture matrix	0.13	−4.7	−10.02	−0.10
Euclidean distance to nearest neighbor	0.13	0.03	−0.01	0.08
Minimum distance to *C. penicillata*	0.11	−0.03	−0.07	0.01
Average size of the forest fragment area	0.08	1.03 × 10^−5^	−1.08 × 10^−5^	7.68 × 10^−5^
Planted forest matrix	0.06	7.38	−25.38	39.70
Flooded areas matrix	0.06	−1.27	−18.57	12.63
Agriculture matrix	0.05	9.54	0	34.95

*Note*: *w*_+_ represents the sum of AICc weights of all models that contain the variable of interest. Variables with *w*_+_ ≥ 0.50 were considered determinants (Berger & Barbieri, 2004). Estimates of variables (*β* parameters) were given by the most parsimonious model that included each variable. CI represents the 95% confidence interval. The *β* parameters are in logit scale.

## 4 DISCUSSION

Landscape composition attributes such as matrix composition and the presence of allochthonous species influence *C. aurita* occurrence within its distribution area in Southeast Brazil. Among the 13 variables considered (Appendix S3), only minimal distance to forest fragments with *C. jacchus* and matrix categories of agriculture and pasture mosaic, nonvegetated, and savanna formation influenced the probability of *C. aurita* occurrence.

Allochthonous species, especially *C. jacchus*, occur near and in hybrid zones together with *C. aurita*. We believe that the presence of *C. jacchus* does not favor the occurrence of *C. aurita*. In fact, the same environmental variables favor the occurrence of both species, which is critical considering the potential for hybridization and genetic introgression between the two (Carvalho et al., 2018; Malukiewicz et al., 2021). Expansions of the distributions of allochthonous species restrict areas of occurrence of *C. aurita*, making it difficult to conserve its genetic integrity (Carvalho et al., 2018). The *Callithrix* genus speciation is relatively recent, which enables hybridization and some natural hybrid zones (Malukiewicz, 2019). Nevertheless, some species would never hybridize naturally because of the distance and barriers (e.g., *C. jacchus* and *C. aurita*; Figure 1). Before human interventions, the six *Callithrix* species had their genetic integrity, but because of trafficking, pet release, and their generalist characteristics (e.g., of *C. penicillata* and *C. jacchus*), some species are expanding and thriving outside their original distribution range (Malukiewicz, 2019). Especially considering that our raw data show 202 registers of *C. jacchus* inside the *C. aurita* distribution area, some of them only 200 m apart from each other, and that the literature shows that they can compete and generate hybrids (e.g., Carvalho et al., 2018; Malukiewicz, 2019), we infer that management actions are urgently needed, given the speed at which the invasive process has occurred (Carvalho et al., 2018). For example, isolated groups of *C. aurita* turned into mixed groups and had hybrid offspring 5 years after *C. jacchus* and *C. penicillata* were introduced in Serra dos Órgãos National Park, state of Rio de Janeiro (Carvalho et al., 2018). Considering that the data of the present study were generated on a time scale of almost 30 years, it is expected that currently only hybrids can be found in some areas. Although pure and isolated populations of *C. aurita* still exist, the advancement of allochthonous species and increased habitat fragmentation and conversion continue to press the species towards extinction (Carvalho et al., 2018; Vital et al., 2020).

Agriculture and pasture mosaic in the surrounding matrix positively influenced the probability of *C. aurita* occurrence. In these regions (Minas Gerais, São Paulo, and Rio de Janeiro), native fragments are largely surrounded by anthropic matrices, and this is part of the history of the Atlantic Forest deforestation (Fonseca, 1985), which ended up isolating species into patches. These matrices are not the most suitable for the movement of arboreal species (Arroyo‐Rodríguez & Mandujano, 2009), but can provide additional resting and feeding areas (Pozo‐Montuy et al., 2013). In addition, this mosaic, when in large dimensions, can also offer resistance to the movement and entry of congeneric allochthonous species, thus favoring the conservation of *C. aurita* within forest fragments. Another factor to be considered is navigation capacity, since the visibility of forest fragments positively influences the probability of *C. aurita* occurrence, as demonstrated by Silva et al. (2015). In the literature, we found that some species of monkey, such as *C. aurita*, have a smaller probability to travel between patches if they can not see the other patch from where it stands (Silva et al., 2015), which means that open areas would favor the movement of individuals of the species and make them occupy these patches.

In contrast to what was expected, savanna formation in the surrounding matrix also showed a positive relationship with *C. aurita* occurrence. This type of environment does not favor the occurrence of arboreal species. Nonetheless, it is possible that the rough indication of the distribution area of the species provided by IUCN (Melo et al., 2021) contains border areas between the Atlantic Forest and the Cerrado. Therefore, there would be areas of Cerrado phyto‐physiognomies interspersed with Atlantic Forest, such as gallery forests, which could favor the occurrence of the species. On the contrary, and as expected, nonvegetated areas in the surrounding matrix had a negative influence on the probability of *C. aurita* occurrence. As these areas do not have canopy formation, they do not provide refuges or feeding areas for arboreal species, nor even favor their movement (Arroyo‐Rodríguez & Mandujano, 2009).

The other variables did not influence the probability of *C. aurita* occurrence. There was just a single record of the species using planted forest in the surrounding matrix (Norris et al., 2011), suggesting that that *C. aurita* may be more selective in the use of the habitat and typically avoids it. On the contrary, both the pasture matrix and the agriculture matrix present less structural heterogeneity when compared to the agriculture and pasture mosaic matrix, and the absence of its influence over *C. aurita* occurrence is corroborated by the study of Silva et al. (2015). There are still large, vegetated areas of forest formation in the landscape, which characterizes it as a non‐limiting variable. Furthermore, these areas can act as stepping‐stones for the native species (Driscoll et al., 2013), but they can also serve as a gateway for allochthonous species (Alharbi & Petrovskii, 2018). Thus, it is essential that the factors that influence the occurrence of allochthonous species be evaluated to help mitigate their immigration.

The lack of an influence of fragment size corroborates Oliveira (2012), who recorded the occurrence of *C. aurita* in fragments of different magnitude of size. Furthermore, considering the relationship between home range and resource availability (Oliveira, 2012), landscape supplementation may be occurring in smaller fragments (Pozo‐Montuy et al., 2013; Valença‐Silva et al., 2014). On the contrary, the ENN metric, despite being very usual, ignores landscape components, which may represent a simplification of the system and, therefore, omit landscape attributes that may be more associated with landscape connectivity for *C. aurita* (Hargis et al., 1998). Altitude, despite being relevant on a refined scale (Norris et al., 2011), does not seem to have the same influence on a landscape scale. *Callithrix aurita* can be found over a large altitudinal range, even though most low‐altitude areas have been decimated by human activities in recent centuries (Brandão & Develey, 1998).

Furthermore, there is a major knowledge gap about *C. aurita's* ability to disperse in different types of matrices. In addition, management, for the control, removal, or another type of strategy specific to each location, of allochthonous species in protected areas is essential for the successful conservation of the genetic integrity of *C. aurita* in situ (Carvalho et al., 2018; ICMBio/MMA, 2018; Malukiewicz, 2019; Moraes et al., 2019). Finally, it is worth noting that for some variables, such as those associated with large estimates of confidence intervals (e.g., nonvegetated areas matrix and savanna formation matrix), more data collection (i.e., species registers) is necessary to increase their strength of inference on whether or not they influence the species occurrence.

Based on the results, we suggest actions that could be integrated into the PAN PPMA, such as monitoring *C. aurita* populations, in order to expand knowledge and update data on the occurrence and environmental predictors of the species (ICMBio/MMA, 2018). We suggest to give priority to the management of allochthonous species and their hybrids since we have found 279 registers of allochthonous species inside the *C. aurita* distribution area, with the aim of conserving the integrity of the native taxon (ICMBio/MMA, 2018). It should be noted that the scope of this work already provides insights that contribute to meet the demands of the PAN PPMA since it expands knowledge about the environmental predictors that act on the occurrence of *C. aurita*. By demonstrating the influence of landscape elements, it becomes evident that factors external to protected areas need to be considered in order to plan with greater assertiveness the next steps *C. aurita* conservation. In addition, this study also provides a basis for the influence of allochthonous species on the probability of *C. aurita* occurrence, which is an alarming finding and supports prioritizing local studies to support decision‐making for the management of allochthonous species. The current scenario may be even more critical in some locations, with the presence of hybrids and even just them, such as in the Rio Doce State Park in Minas Gerais, where only one group with *C. aurita* individuals were found, and all of the other groups we composed by hybrids (Guimarães‐Lopes et al., Unpublished data). This means that we are losing the pure genetics of an already threatened species because of the biological invasions.

We hope that the results of this work can also contribute substantially to the conservation of other primate species that face the same challenges as *C. aurita* and are endangered, as is the case of *C. flaviceps*. This is possible once they can support the definition of priority areas to implement conservation plans for those primates, such as habitat restoration, and implementation of corridors between patches, also showing the necessity of new population surveys and censuses in some areas. All of these are in order to understand where we need to put the effort in rescuing the last pure individuals to ex situ management, and also do something about the biological invasions, with the purpose of preventing new colonization and the genetic mix between native and invasive species.

## AUTHOR CONTRIBUTIONS

**Natasha Grosch Loureiro:** Conceptualization (equal); data curation (lead); formal analysis (lead); investigation (equal); methodology (equal); software (lead); writing – original draft (lead). **Vanessa de Paula Guimarães‐Lopes:** Conceptualization (equal); investigation (supporting); methodology (equal); writing – original draft (supporting); writing – review and editing (supporting). **Flávio Henrique Guimarães Rodrigues:** Conceptualization (equal); methodology (equal); supervision (lead); writing – review and editing (equal). **Rodrigo Lima Massara:** Conceptualization (equal); methodology (equal); software (equal); supervision (equal); writing – review and editing (equal).

## FUNDING INFORMATION

Coordenação de Aperfeiçoamento de Pessoal de Nível Superior – CAPES (Grant 88887.342104/2019‐00).

## CONFLICT OF INTEREST STATEMENT

The authors declare that they have no known competing financial interests or personal relationships that could have appeared to influence the work reported in this paper.

## Supporting information


Appendix S1–S3

## Data Availability

The data that support the findings of this study are openly available in Dryad at https://doi.org/10.5061/dryad.8sf7m0ctc.
