# Supplementary material for: Landscape factors and allochthonous congeneric species influence Callithrix aurita occurrence in Brazilian Atlantic Forest remnants
Source: Ecol Evol. 2023 Apr 7;13(4):e9968. doi: 10.1002/ece3.9968 (PMC10082169; doi:10.1002/ece3.9968)

**Appendix**

**Appendix S1:** Database used for the analysis, containing identification (id), coordinates of the centroids (in WGS84; latitude and longitude), presence, number of records (registers), area (in hectares), altitude (in meters), enn index (enn), proximity index (prox), average and minimum distances for *C. penicillata* and *C. jacchus* (in meters; d_med and d_min), and the matrices (mat_3: forest formation, mat_4: savanna formation, mat_9: planted forest, mat_15: pasture, mat_21: agriculture and pasture mosaic, mat_a_n_veg: non-vegetated areas, mat_a_alag: flooded areas and, mat_agricu: agriculture).

| id | latitude | longitude | presence | registers | area | altitude | Enn | prox | d_med_pen | d_min_pen | d_med_jac | d_min_jac | mat_3 | mat_4 | mat_9 | mat_15 | mat_21 | mat_a_n_veg | mat_a_alag | mat_agricu |
| --- | --- | --- | --- | --- | --- | --- | --- | --- | --- | --- | --- | --- | --- | --- | --- | --- | --- | --- | --- | --- |
| AP_11 | -42.469 | -22.013 | s | 2 | 271.68 | 558.95 | 65 | 762.4 | 239.31 | 49.82 | 242.4 | 49.77 | 0.3581 | 0.0002 | 0.0105 | 0.4671 | 0.1571 | 0.0016 | 0.0022 | 0 |
| AP_12 | -42.460 | -22.075 | s | 2 | 5900.03 | 802.51 | 60.03 | 1672.82 | 239.28 | 44.49 | 240.15 | 44.44 | 0.2473 | 0.001 | 0.0097 | 0.4291 | 0.2034 | 0.0039 | 0.0005 | 0 |
| AP_17 | -43.351 | -22.248 | s | 1 | 7.74 | 439.14 | 94.81 | 11.9 | 190.62 | 21.11 | 192.48 | 34.07 | 0.1213 | 0.0001 | 0.001 | 0.6921 | 0.1746 | 0.0031 | 0.0046 | 0 |
| AP_18 | -45.275 | -22.597 | s | 1 | 350958.01 | 1367.13 | 60 | 68490.53 | 218.74 | 0 | 214.5 | 17.85 | 0.2159 | 0.0001 | 0.0638 | 0.3985 | 0.2746 | 0.0111 | 0.0046 | 0.0022 |
| AP_21 | -42.563 | -19.694 | s | 1 | 46708.36 | 298.89 | 59.97 | 11261.85 | 350.17 | 0 | 403.69 | 61.7 | 0.28 | 0.05 | 0.11 | 0.27 | 0.13 | 0.03 | 0.07 | 0.0044 |
| AP_22 | -42.737 | -20.066 | s | 2 | 66.61 | 417.66 | 111.9 | 65.56 | 313.12 | 45.09 | 364.53 | 59.54 | 0.198 | 0.082 | 0.001 | 0.6026 | 0.0515 | 0.0005 | 0.0451 | 0.0188 |
| AP_29 | -46.659 | -23.167 | s | 1 | 26.43 | 874.63 | 107.8 | 127.57 | 290.47 | 16.34 | 272.94 | 14.23 | 0.2948 | 0 | 0.0126 | 0.2435 | 0.4017 | 0.01 | 0.0043 | 0.01 |
| AP_30 | -46.171 | -23.453 | s | 1 | 8566.89 | 791.25 | 60.02 | 2764.07 | 275.79 | 6.17 | 243.48 | 0.76 | 0.2385 | 0 | 0.0145 | 0.2021 | 0.3225 | 0.0472 | 0.0027 | 0.0005 |
| AP_31 | -44.749 | -22.917 | s | 1 | 18.24 | 1361.41 | 68.31 | 3194.93 | 211.84 | 58.55 | 194.37 | 29.69 | 0.5381 | 0 | 0.0256 | 0.2512 | 0.1824 | 0.0025 | 0 | 0.0003 |
| AP_34 | -44.350 | -22.786 | s | 1 | 874.83 | 1375.55 | 57.29 | 166.14 | 200.92 | 42.36 | 183.59 | 47.56 | 0.7712 | 0 | 0.0051 | 0.1366 | 0.0859 | 0.0009 | 0 | 0.0003 |
| AP_37 | -42.928 | -20.734 | s | 1 | 135.79 | 740.02 | 72.51 | 83.18 | 261.15 | 60.5 | 304.23 | 75.99 | 0.236 | 0 | 0.0033 | 0.5754 | 0.1289 | 0.0187 | 0.0002 | 0 |
| AP_38 | -46.546 | -23.131 | s | 1 | 31.39 | 802.66 | 71.4 | 39.2 | 282.18 | 5.31 | 265.26 | 20.72 | 0.2776 | 0 | 0.0032 | 0.1002 | 0.3101 | 0.1 | 0.0022 | 0.001 |
| AP_39 | -46.969 | -23.717 | s | 1 | 14112.99 | 945.17 | 57.42 | 10569.27 | 336.93 | 11.05 | 308.22 | 1.22 | 0.2869 | 0 | 0.0092 | 0.0788 | 0.4593 | 0.0493 | 0.012 | 0.003 |
| AP_40 | -46.565 | -23.154 | s | 1 | 56.3 | 809.57 | 57.94 | 367.61 | 284.07 | 8.54 | 266.35 | 19.07 | 0.2884 | 0 | 0.0041 | 0.1015 | 0.3313 | 0.089 | 0.0016 | 0.0021 |
| AP_42 | -46.498 | -23.312 | s | 4 | 64208.61 | 921.84 | 56.46 | 31130.72 | 285.93 | 23.94 | 261.33 | 1.76 | 0.268 | 0 | 0.0343 | 0.0969 | 0.3142 | 0.0665 | 0.0178 | 0.0007 |
| AP_43 | -46.358 | -23.207 | s | 1 | 79.83 | 828.59 | 57.98 | 2764.7 | 274.62 | 19.9 | 252.65 | 16.92 | 0.4226 | 0 | 0.0707 | 0.11 | 0.25 | 0.0034 | 0.14 | 0.0005 |
| AP_63 | -46.356 | -23.195 | s | 1 | 88.13 | 841.17 | 57.3 | 2941.9 | 274.15 | 19.25 | 252.64 | 17.94 | 0.407 | 0 | 0.0742 | 0.1198 | 0.2349 | 0.0055 | 0.1446 | 0.0015 |
| AP_67 | -42.453 | -20.693 | s | 1 | 22787.33 | 1326.09 | 58.77 | 6248.35 | 284.9 | 54.12 | 322.75 | 26.77 | 0.2245 | 0.0009 | 0.0165 | 0.4987 | 0.254 | 0.0019 | 0 | 0 |
| AP_68 | -42.765 | -22.438 | s | 1 | 361270.79 | 749.43 | 56 | 52585.09 | 217.31 | 0.22 | 207.77 | 0.22 | 0.2411 | 0 | 0.0037 | 0.4561 | 0.2413 | 0.0187 | 0.0012 | 0.0004 |
| AP_71 | -45.057 | -22.110 | s | 1 | 14.65 | 905.47 | 115.12 | 6.41 | 211.7 | 16.1 | 222.82 | 66.09 | 0.1551 | 0 | 0.001 | 0.53 | 0.2061 | 0.0469 | 0.0015 | 0.0063 |
| AP_72 | -45.423 | -21.970 | s | 1 | 5492.02 | 1169.66 | 56.21 | 5742.64 | 229.14 | 47.69 | 243.59 | 86.03 | 0.2023 | 0 | 0.0137 | 0.4397 | 0.3106 | 0.0029 | 0.0036 | 0.0071 |
| AP_74 | -47.202 | -23.285 | s | 1 | 17.31 | 730.66 | 57.22 | 1486.61 | 336.82 | 26.72 | 318.46 | 19.77 | 0.3147 | 0 | 0.0841 | 0.3104 | 0.2662 | 0.0047 | 0.0087 | 0.0022 |
| AP_75 | -45.053 | -23.254 | s | 1 | 75.02 | 1181.66 | 57.15 | 2471.44 | 231.42 | 31.9 | 206.88 | 26.84 | 0.7315 | 0 | 0.0117 | 0.1763 | 0.0789 | 0.0007 | 0 | 0.0018 |
| FS_30 | -43.079 | -21.542 | n | 0 | 793.42 | 641.1 | 57.6 | 325.36 | 217.31 | 53.36 | 241.72 | 103.06 | 0.1038 | 0 | 0.0017 | 0.7905 | 0.0828 | 0.0103 | 0.0006 | 0 |
| FS_31 | -44.089 | -22.553 | n | 0 | 291.79 | 440.63 | 61.25 | 391.26 | 191.98 | 43.96 | 180.05 | 18.12 | 0.1499 | 0 | 0.0002 | 0.3984 | 0.1873 | 0.1211 | 0.022 | 0 |
| FS_32 | -43.501 | -22.811 | n | 0 | 6346.31 | 438.56 | 56.95 | 495.75 | 199.67 | 9.91 | 173.81 | 10.38 | 0.0524 | 0 | 0 | 0.1982 | 0.1473 | 0.301 | 0.0002 | 0 |
| FS_33 | -45.997 | -22.946 | n | 0 | 293.23 | 998.35 | 57.31 | 7732.42 | 251.93 | 44.32 | 237.66 | 45.52 | 0.5418 | 0 | 0.0384 | 0.2426 | 0.1741 | 0.0002 | 0.0023 | 0 |
| FS_34 | -45.539 | -23.154 | n | 0 | 274.33 | 802.07 | 64.38 | 39.1 | 240.28 | 14.7 | 219.6 | 62.22 | 0.2465 | 0 | 0.0319 | 0.5731 | 0.1465 | 0.0003 | 0 | 0.0013 |
| FS_35 | -42.085 | -20.813 | n | 0 | 280.59 | 722.16 | 59.51 | 171.83 | 300.83 | 35.2 | 332.06 | 15.3 | 0.2204 | 0 | 0.0059 | 0.6323 | 0.14 | 0.0005 | 0.0006 | 0 |
| FS_8 | -43.091 | -20.812 | n | 0 | 24.69 | 681.67 | 57.82 | 1283.32 | 250.7 | 51.8 | 294.21 | 93.54 | 0.2795 | 0 | 0.0084 | 0.584 | 0.1263 | 0.0002 | 0.0014 | 0 |
| FS_9 | -43.559 | -20.484 | n | 0 | 4.41 | 1105.61 | 66.17 | 376.1 | 260.56 | 12.23 | 314.68 | 144.32 | 0.3802 | 0.0239 | 0.0264 | 0.0946 | 0.2694 | 0.0608 | 0.023 | 0 |
| FS_36 | -42.921 | -20.552 | n | 0 | 277.2 | 654.56 | 59.22 | 1911.72 | 272.35 | 54.88 | 318.78 | 77.7 | 0.3193 | 0 | 0.0179 | 0.4904 | 0.1626 | 0.0007 | 0.0076 | 0 |
| FS_37 | -41.089 | -21.396 | n | 0 | 1182.87 | 31.18 | 164.91 | 3 | 262.85 | 133.9 | 373.98 | 129.29 | 0.0089 | 0 | 0 | 0.2601 | 0.0824 | 0.0006 | 0.0039 | 0.3215 |
| FS_38 | -43.671 | -22.965 | n | 0 | 144.77 | 9.02 | 59.15 | 116.8 | 205.51 | 26.59 | 174.35 | 1.21 | 0.105 | 0 | 0 | 0.1505 | 0.1424 | 0.1942 | 0.0036 | 0 |
| FS_39 | -45.860 | -23.262 | n | 0 | 96.67 | 630.33 | 57.35 | 40.95 | 255.41 | 28.15 | 230.78 | 27.28 | 0.1194 | 0 | 0.0343 | 0.1948 | 0.2326 | 0.1374 | 0.0033 | 0.0011 |
| FS_15 | -41.799 | -21.853 | n | 0 | 43166.01 | 679.95 | 57.2 | 9581.21 | 293.6 | 69.76 | 297.07 | 62.68 | 0.2134 | 0 | 0.0008 | 0.5642 | 0.2071 | 0.0003 | 0.001 | 0.0035 |
| FS_40 | -43.837 | -21.942 | n | 0 | 19165.57 | 1091.09 | 56.79 | 12779.86 | 190.66 | 50.63 | 204.71 | 59.07 | 0.3373 | 0.0015 | 0.0113 | 0.4261 | 0.2157 | 0.0026 | 0.0004 | 0 |
| FS_41 | -43.575 | -21.147 | n | 0 | 468.51 | 1088.59 | 57.66 | 837.44 | 220.1 | 38.65 | 260.86 | 148.81 | 0.2075 | 0 | 0.0117 | 0.6418 | 0.1372 | 0.0005 | 0 | 0.0002 |
| FS_42 | -44.138 | -21.083 | n | 0 | 1645.67 | 1052.67 | 57.24 | 577.14 | 221.06 | 7.97 | 266.03 | 145.76 | 0.1526 | 0.0026 | 0.0096 | 0.4757 | 0.2472 | 0.0216 | 0.0024 | 0.0233 |
| FS_43 | -45.562 | -23.322 | n | 0 | 295.37 | 791.69 | 57.08 | 1317.45 | 247.91 | 32.9 | 222.45 | 56.25 | 0.353 | 0 | 0.0685 | 0.2592 | 0.1354 | 0.0008 | 0.1772 | 0.0043 |
| FS_44 | -42.159 | -22.823 | n | 0 | 436.52 | 113.04 | 61.2 | 152.62 | 274.32 | 43.87 | 249.1 | 27.38 | 0.0719 | 0 | 0 | 0.4384 | 0.0635 | 0.0724 | 0.2812 | 0 |
| FS_45 | -42.277 | -21.060 | n | 0 | 24.34 | 458.04 | 141.35 | 63.97 | 278.27 | 68.4 | 306.26 | 38.04 | 0.2301 | 0 | 0.0118 | 0.669 | 0.0875 | 0.0001 | 0.0013 | 0 |
| FS_46 | -43.139 | -21.154 | n | 0 | 81.2 | 757.93 | 57.78 | 142.04 | 231.29 | 64.04 | 267.3 | 108.85 | 0.2313 | 0 | 0.0036 | 0.6721 | 0.0918 | 0.0001 | 0.0007 | 0 |
| FS_25 | -42.032 | -22.538 | n | 0 | 1600.51 | 268.16 | 57.51 | 12.9 | 276.25 | 10.16 | 259.77 | 26.61 | 0.1144 | 0 | 0 | 0.6527 | 0.0952 | 0.0319 | 0.0119 | 0.0182 |
| FS_47 | -45.892 | -22.684 | n | 0 | 34.55 | 1614.86 | 57.46 | 2021.57 | 244.35 | 50 | 238.14 | 76.44 | 0.4556 | 0 | 0.0131 | 0.3067 | 0.2217 | 0.0009 | 0 | 0.0002 |
| FS_48 | -44.764 | -21.934 | n | 0 | 28.41 | 1063.45 | 67.15 | 149.05 | 204.58 | 19.2 | 221.71 | 81.71 | 0.294 | 0.0049 | 0.0115 | 0.5056 | 0.1761 | 0.0024 | 0 | 0.0016 |

**Appendix S2:** R code for the data analysis.

#Packages

library(AICcmodavg)

library(sjPlot)

#Input

land = read.table("aurita_matrizcat_vec.txt", header = T, dec = ",")

View(land)

summary(land)

#Correlation test - Pearson

cor(land[,c(4,5,6,7,8,9,10,11,12,13,14,15,16,17,18,19)])

#Doing the balanced models

library (MuMIn)

library(stats)

#All of the combinations

full<- glm(presence~area+altitude+enn+d_min_pen+d_min_jac+mat_3+mat_4+mat_9+mat_15+mat_21+mat_a_n_veg+mat_a_alag+mat_agricu, family=binomial(link="logit"), data=land2)

options(na.action = "na.fail")

fm1<-dredge(full, m.lim = c(0,4))

selec <- dredge(full, rank="AICc", extra = "adjR^2", m.lim = c(0,4))

fix(selec)

##check c-hat for global model

#with simulation - 10000

install.packages("devtools")

library("devtools")

install.packages("DHARMa")

library("DHARMa")

require(DHARMa)

sim_mod1 <- simulateResiduals(full, refit=F, n=10000)

testDispersion(sim_mod1)

plot(sim_mod1)

##compute importance (cumulative weight - w+)

importance(fm1)

#Moran’s test

res = simulateResiduals(full)

testSpatialAutocorrelation(res, x = land$latitude, y = land$longitude)

#CI of the most parcimonious models containing the variables

#Variables: d_min_jac; mat_21; mat_4; mat_a_n_veg

modelo1 = glm(presence~d_min_jac+ mat_21+ mat_4+ mat_a_n_veg, family = binomial (link="logit"), data = land2)

summary(modelo1)

cbind(coef(modelo1), confint(modelo1))

#Variable: mat3

modelo2 = glm(presence~d_min_jac+ mat_21+ mat_3+ mat_4, family = binomial (link="logit"), data = land2)

summary(modelo2)

cbind(coef(modelo2), confint(modelo2))

#Variable: average altitude

modelo3 = glm(presence~d_min_jac+ mat_21+ mat_4+ altitude, family = binomial (link="logit"), data = land2)

summary(modelo3)

cbind(coef(modelo3), confint(modelo3))

#Variable: enn

modelo7 = glm(presence~d_min_jac+ enn+ mat_4+ altitude, family = binomial (link="logit"), data = land2)

summary(modelo7)

cbind(coef(modelo7), confint(modelo7))

#Variable: pasture

modelo8 = glm(presence~d_min_jac+ mat_15+ mat_4+ mat_a_n_veg, family = binomial (link="logit"), data = land2)

summary(modelo8)

cbind(coef(modelo8), confint(modelo8))

#Variable: C. penicillata

modelo9 = glm(presence~d_min_jac+ d_min_pen+mat_21+mat_a_n_veg, family = binomial (link="logit"), data = land2)

summary(modelo9)

cbind(coef(modelo9), confint(modelo9))

#Variable: fragment size

modelo17 = glm(presence~ area+ d_min_jac+ mat_21+mat_a_n_veg, family = binomial (link="logit"), data = land2)

summary(modelo17)

cbind(coef(modelo17), confint(modelo17))

#Variable: planted matrix

modelo21 = glm(presence~ d_min_jac+ mat_21+ mat_9+ mat_a_n_veg, family = binomial (link="logit"), data = land2)

summary(modelo21)

cbind(coef(modelo21), confint(modelo21))

#Variable: flooded areas

modelo24 = glm(presence~ d_min_jac+ mat_21+ mat_a_alag+ mat_a_n_veg, family = binomial (link="logit"), data = land2)

summary(modelo24)

cbind(coef(modelo24), confint(modelo24))

#Variable: matrix agriculture

modelo25 = glm(presence~ altitude+ d_min_jac+ mat_4+ mat_agricu, family = binomial (link="logit"), data = land2)

summary(modelo25)

cbind(coef(modelo25), confint(modelo25))

##Figure’s code example

ggplot(land, aes (x=mat_3, y=presence, color = as.factor(presence))) + geom_point(size = 2) + geom_smooth(method = "glm", se = T, method.args = list(family = "binomial"), linetype = 1, col = 1, size = 0.7) + labs(x= expression("Forest formation matrix (%)"), y= NULL) + theme(axis.text = element_text(color="black", size = 10), axis.text.y = element_text(angle = 90, hjust = 0.5), panel.background = element_rect(fill = F), axis.line = element_line(size = 0.5, colour = "black", linetype=1), axis.title.y = element_text(margin = margin(t = 0, r = 15, b = 0, l = 0), size = 10), axis.title.x = element_text( size = 10), legend.title = element_blank(), legend.position = "none") + scale_y_continuous(labels=function(x) format(x, decimal.mark = ".", scientific = F)) + scale_x_continuous(labels= function(x) paste0(x*100)) + scale_color_manual(values=c("dimgray","black"), labels = c("Controle", expression(paste(italic("C. aurita")))))

**Appendix S3:** Relation of the variables that did not influence the probability of *C. aurita* occurrence in forest fragments that occur within the species' distribution in Southeast Brazil according to the model selection theory (i.e., accumulative AICc weight; *w_+_*) adopted by us: (1) average area size, (2) average altitude, (3) forest formation matrix, (4) planted forest matrix, (5) pasture matrix, (6) flooded areas matrix, (7) agriculture matrix, (8) Minimum distance to *C. penicillata*, and (9) Euclidean distance to nearest neighbor (ENN). Black dots represent fragments with the confirmed occurrence of *C. aurita* while gray dots represent those without (control). The grey range represents the confidence intervals.

1. Average area size:


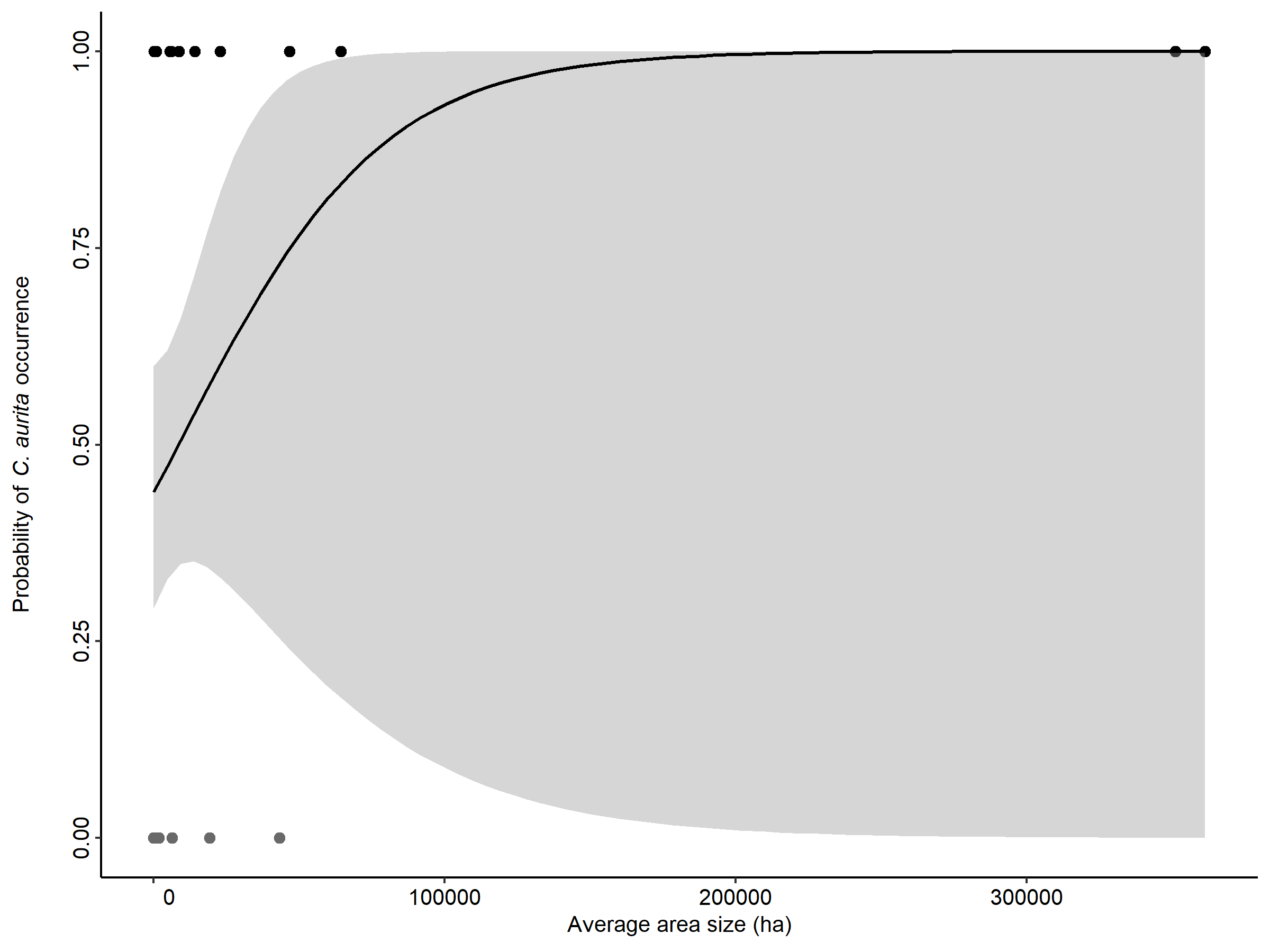


1. Average altitude:


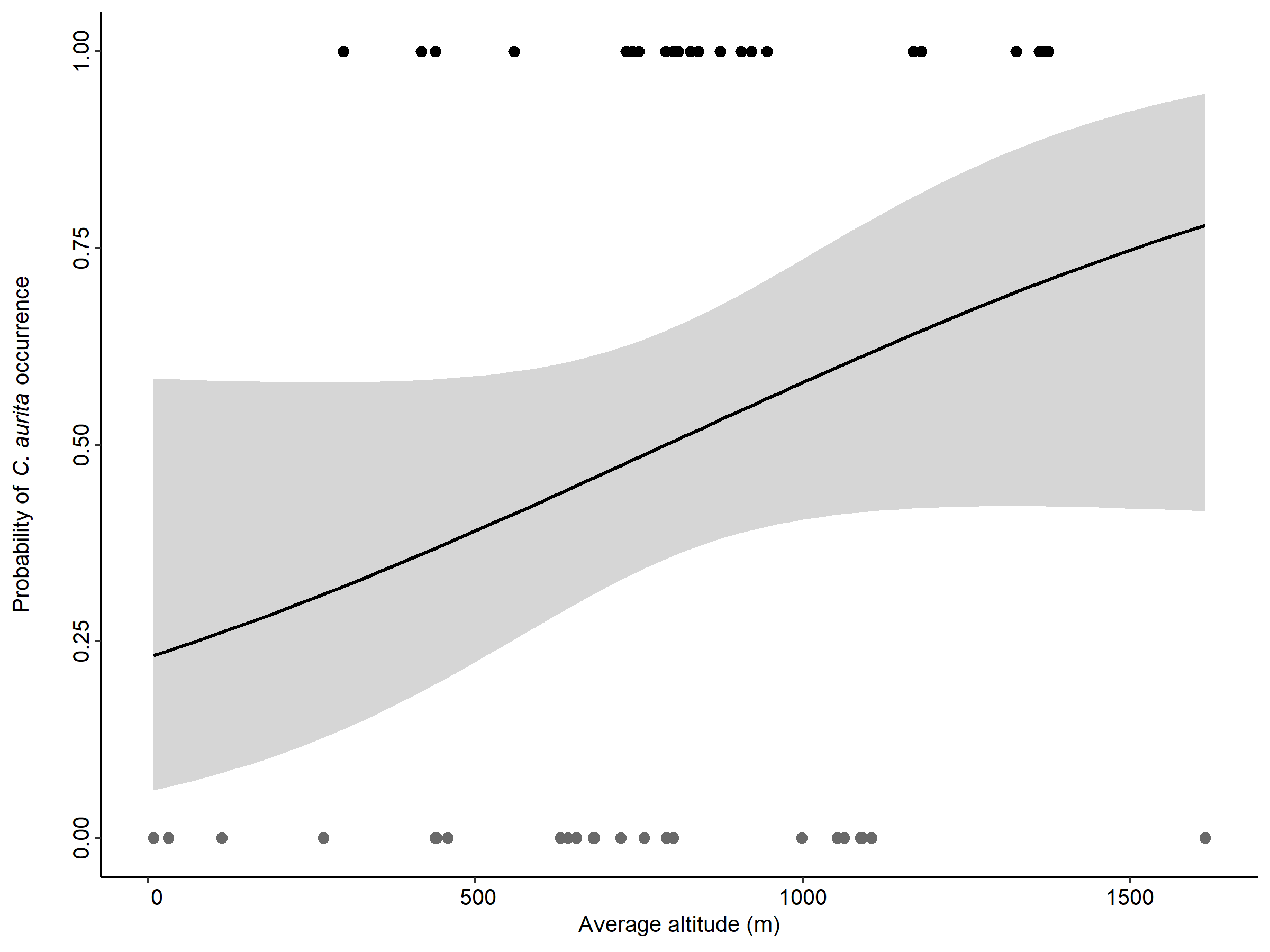


1. Forest formation matrix:


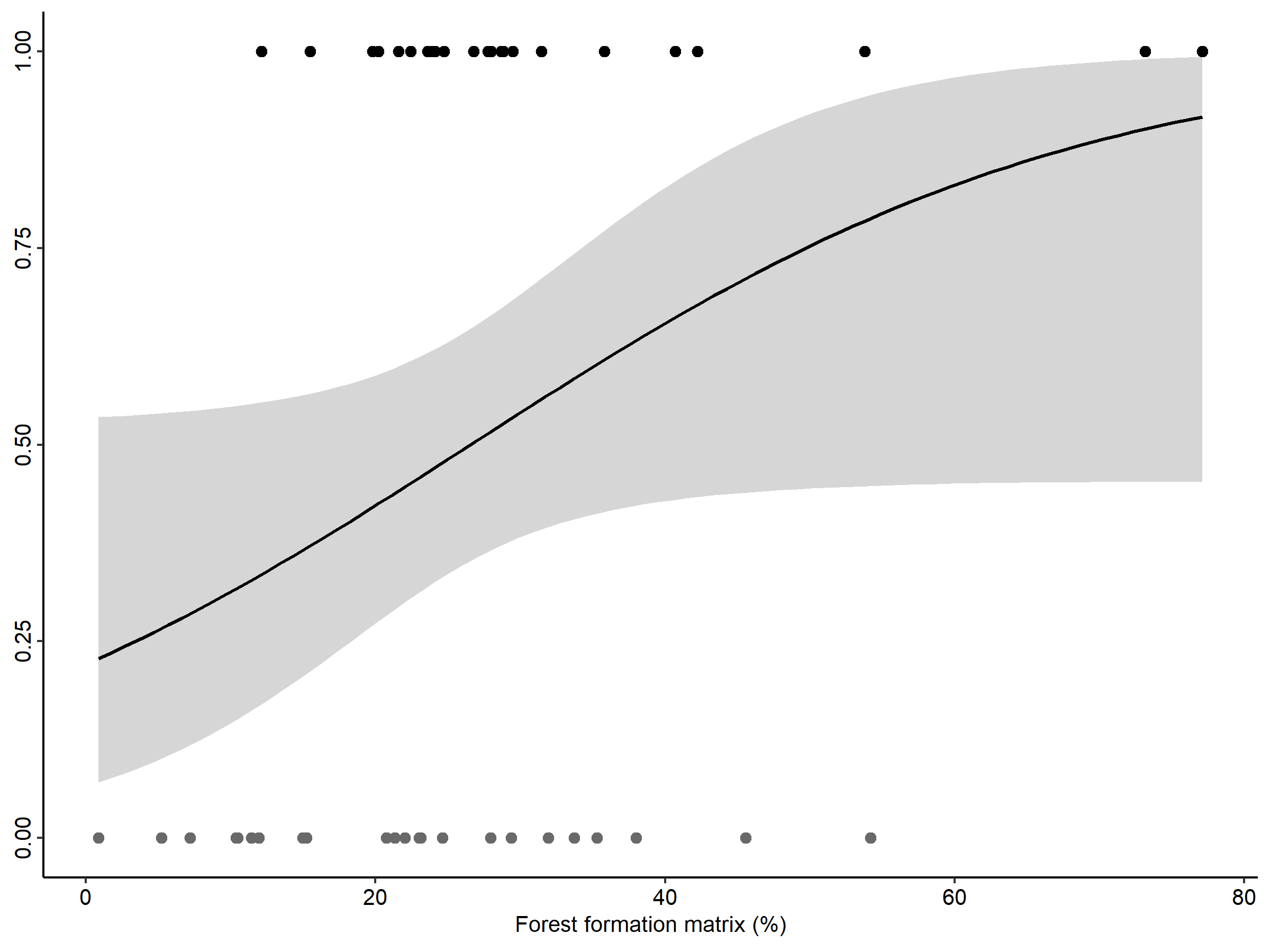


1. Planted forest matrix:


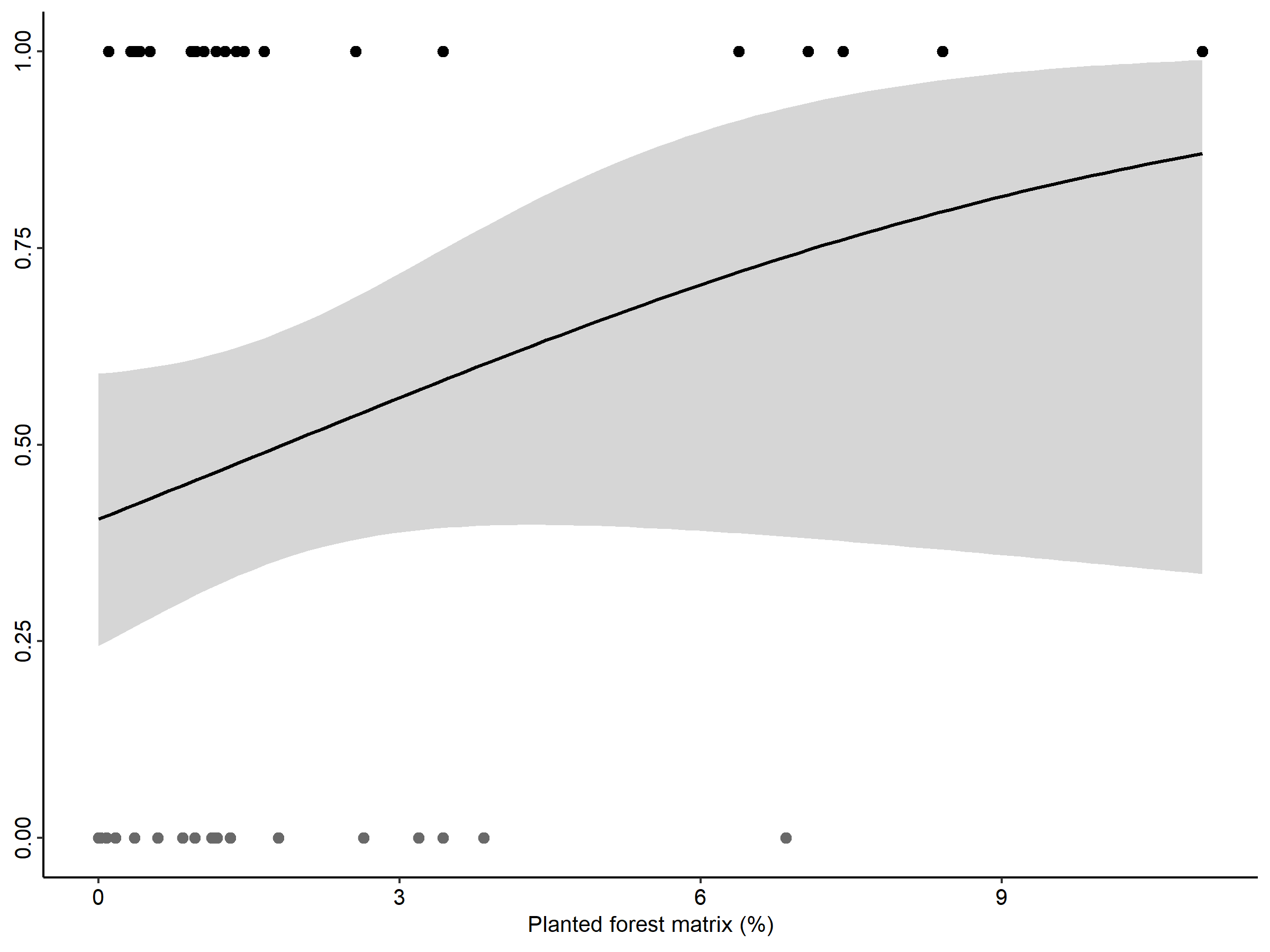


1. Pasture matrix:


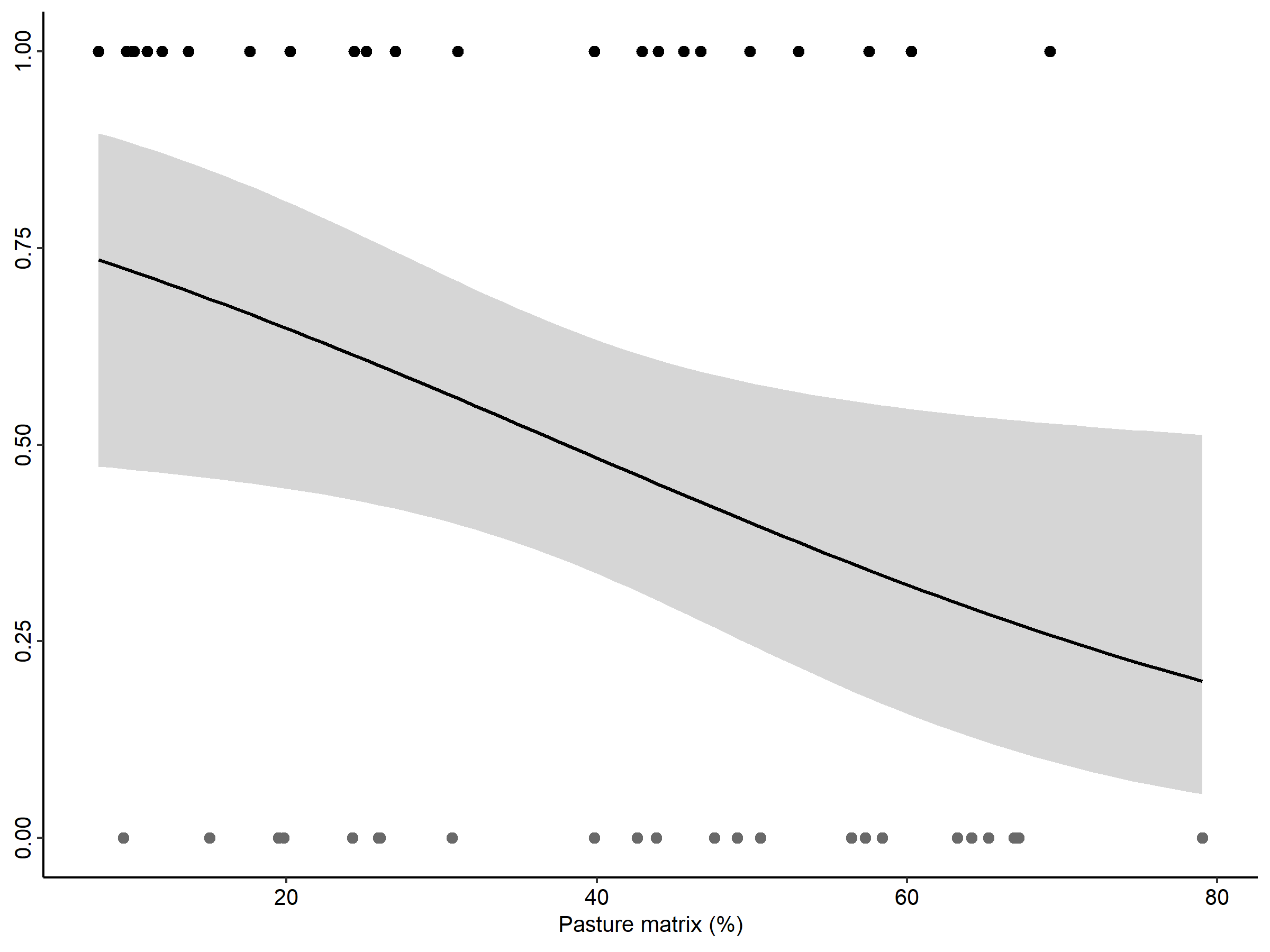


1. Flooded areas matrix:


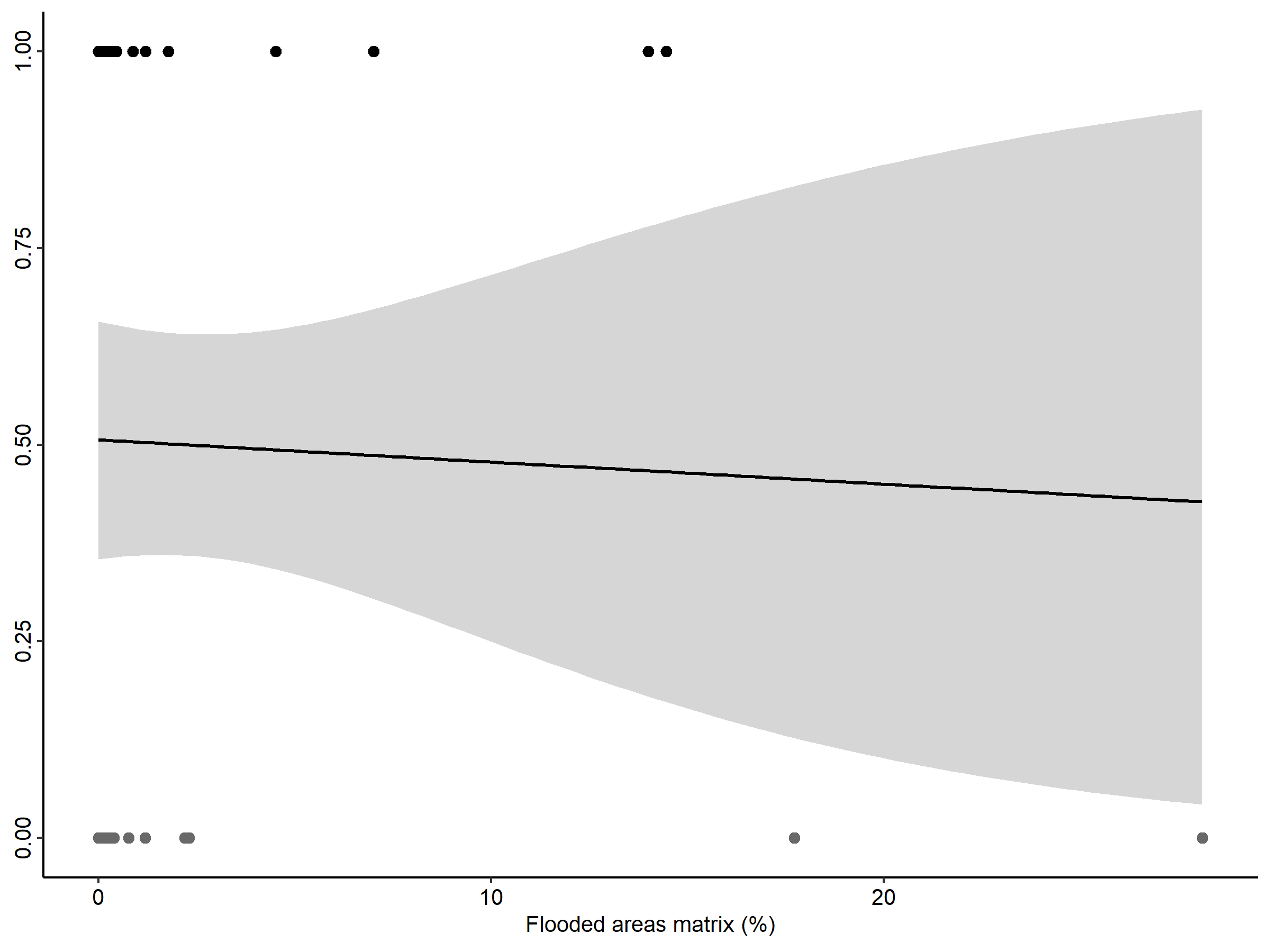


1. Agriculture matrix:


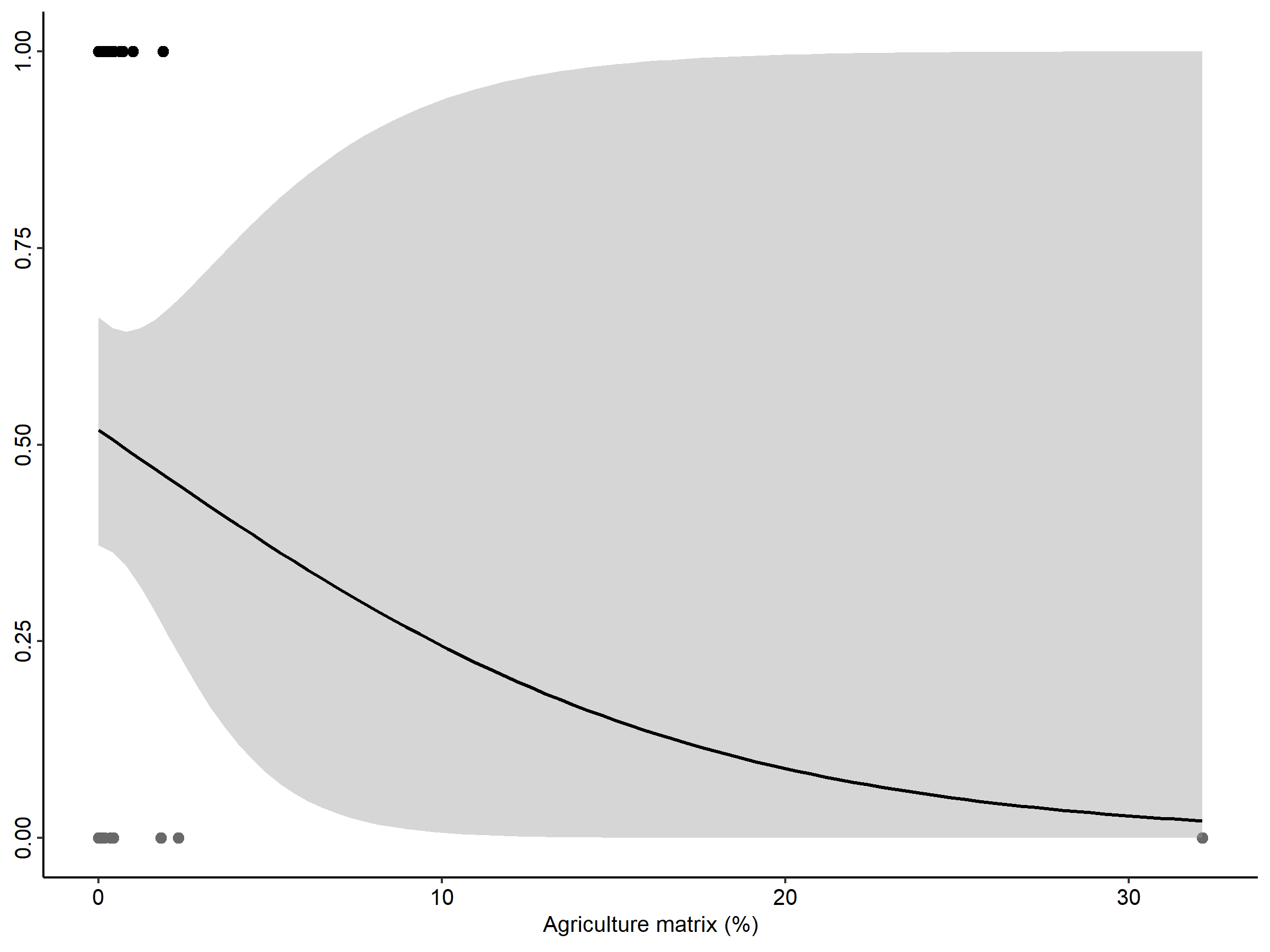


1. Minimum distance to *C. penicillata*:


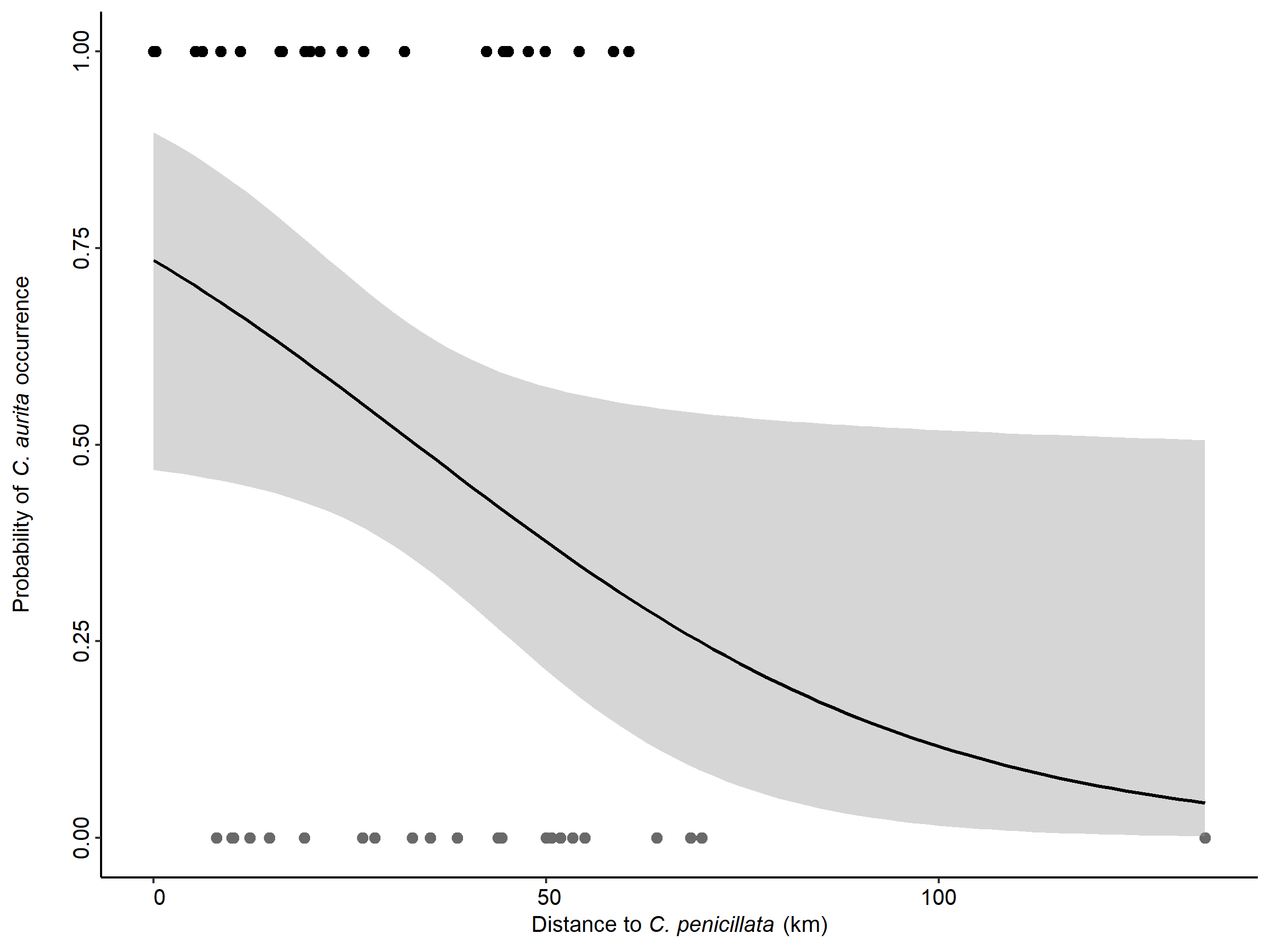


1. Euclidean distance to nearest neighbor (ENN):


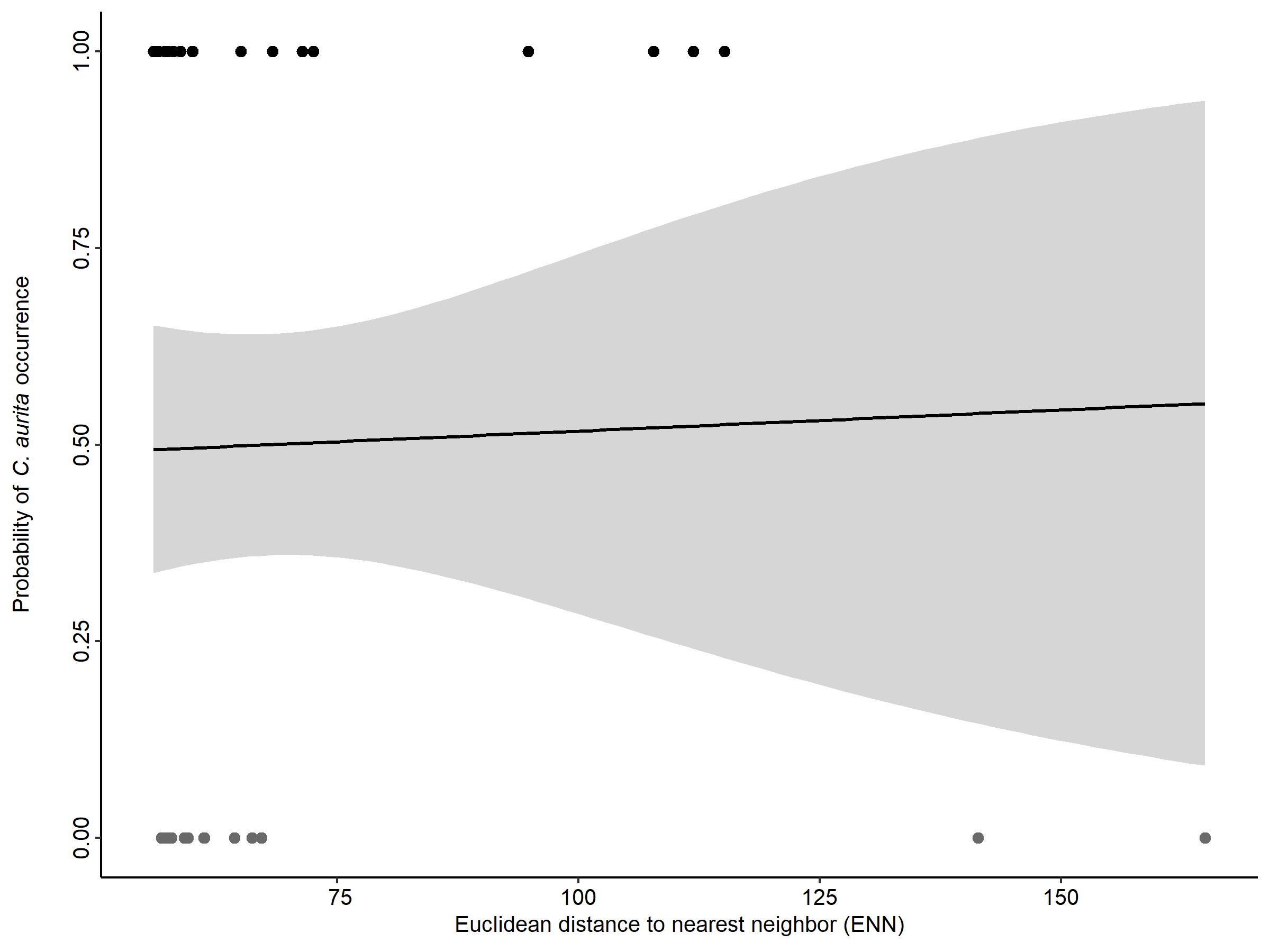

Supplement: Supplementary file 1 — Appendix S1–S3 [file ECE3-13-e9968-s001.docx]
